# The use of esketamine in comorbid treatment resistant depression and obsessive compulsive disorder following extensive pharmacogenomic testing: a case report

**DOI:** 10.1186/s12991-021-00365-z

**Published:** 2021-09-16

**Authors:** Marcatili Matteo, Pellicioli Cristian, Maggioni Laura, Motta Federico, Redaelli Chiara, Ghelfi Lorenzo, Krivosova Michaela, Matteo Sibilla, Nava Roberto, Colmegna Fabrizia, Dakanalis Antonios, Caldiroli Alice, Capuzzi Enrico, Benatti Beatrice, Bertola Francesca, Villa Nicoletta, Piperno Alberto, Ippolito Silvia, Clerici Massimo

**Affiliations:** 1 Psychiatric Department, San Gerardo Hospital, ASST Monza, Monza, Italy; 2 Department of Medicine and Surgery, University of Milano Bicocca, Monza, Italy; 3 Faculty of Medicine, University Vita-Salute San Raffaele, Milan, Italy; 4 Department of Pharmacology, Jessenius Faculty of Medicine in Martin, Comenius University in Bratislava, Martin, Slovakia; 5 Psychiatry Unit, Department of Biomedical and Clinical Sciences “Luigi Sacco”, University of Milan, Milan, Italy; 6 CRC “Aldo Ravelli” for Neurotechnology and Experimental Brain Therapeutics, University of Milan, Milan, Italy; 7 Cytogenetics and Medical Genetics Unit, Centre for Disorders of Iron Metabolism, San Gerardo Hospital, ASST Monza, Monza, Italy; 8 Clinical Chemistry Laboratory, San Gerardo Hospital, ASST Monza, Monza, Italy

**Keywords:** Treatment resistant depression, TRD, Obsessive–compulsive disorder, OCD, Esketamine, Ketamine, SLC6A4, MTHFR, GABRP, GABRA6

## Abstract

**Background:**

Major depressive disorder (MDD) patients not responding to two or more different antidepressant treatments are currently considered to suffer from treatment resistant depression (TRD). Recently, intranasal esketamine has been approved by both the American Food and Drug Administration and European Medicines Agency for TRD and, more recently, in moderate to severe episode of MDD, as acute short-term treatment for the rapid reduction of depressive symptoms, which, according to clinical judgement, constitute a psychiatric emergency. There is currently no indication for obsessive–compulsive disorder (OCD) although recently published studies have already shown a rapid and significant reduction of OCD-like symptoms following ketamine administration. The etiology of OCD has not yet been fully elucidated but there is a growing evidence that glutamate signaling dysfunction in the cortico-striatal–thalamo-cortical circuitry plays an essential role. This case report exemplifies possible clinical effects of esketamine on both depressive and OCD symptoms.

**Case presentation:**

We present the case of a 39-year-old man suffering from TRD. During the first evaluation at our clinic, he also reported the presence of OCD spectrum symptoms, causing him to perform time-consuming mental rituals due to pathological doubts regarding the relationship with his wife as well as intrusive thoughts regarding his mental conditions. He underwent psychometric evaluations, therapeutic drug monitoring analysis, and pharmacogenomic tests. The overall results helped to explain patient’s treatment-resistance. Moreover, we observed a significant reduction in both depressive and OCD symptoms after administration of esketamine.

**Conclusion:**

This case underlines the importance of pharmacogenomic tests in profiling TRD patients and confirms the possible use of esketamine in the treatment of comorbid OCD.

## Background

Major depressive disorder (MDD) is a common mental disorder and a leading cause of disability, affecting more than 264 million people worldwide [1, 2]. The STAR*D study demonstrated that only one third of patients achieved remission following the first antidepressant treatment and, even after 1 year of therapy with a sequence of four antidepressants administered for 12 weeks each, only two-third of patients achieved symptoms remission [3]. Although no single definition of treatment resistant depression (TRD) exists, it generally indicates patients who failed to respond to two or more trials of antidepressants, at adequate dosage and treatment duration [4]. As TRD patients seem not to respond sufficiently to traditional monoaminergic antidepressants, new treatment strategies acting on glutamatergic, cholinergic, and opioid systems are currently under investigation [5]. Pharmacogenomic testing (PGx) represents a decision-support tool that has been recently introduced into the clinical practice in psychiatry. Such personalized approach is especially useful in patients with conditions resistant to standard treatments due to genetic predisposition to poor psychopharmacological response or high susceptibility to severe side effects. PGx has several benefits: it could both lower the latency to clinical response or remission and increase patient’s compliance by reducing side effects impact and cost-effectiveness of the whole clinical management. The comorbidity of depression with other psychiatric disorders has been described in the past, and one of the most common comorbidities is represented by Obsessive–Compulsive Disorder (OCD) [6–8]. The coexistence of the two disorders seems to lead to a greater symptoms severity, less satisfactory response to treatment and an overall less favorable prognosis [8]. The disorders share common psychopathological characteristics and, in some cases, also treatment response [9–11]. Dysregulation of glutamate signaling in the cortico-striatal–thalamo-cortical circuitry appears to play a role in OCD as supported by preclinical, neuroimaging, and genetic studies [12–18].

Intranasal esketamine has been approved by both the American Food and Drug Administration and the European Medicines Agency for TRD in adults and, more recently, in moderate to severe episode of MDD, as acute short-term treatment for the rapid reduction of depressive symptoms, which according to clinical judgement constitute a psychiatric emergency. Esketamine is the (S) enantiomer of ketamine, a non-competitive *N*-methyl-d-aspartate (NMDA) glutamate receptor antagonist that was introduced in clinics as an anesthetic and analgesic more than 50 years ago [19, 20]. The mechanism of antidepressant action of esketamine has not been fully clarified yet but modulation of different signaling pathways implicated in the pathophysiology of MDD, such as synaptogenesis and neuroplasticity pathways, may play a role [21, 22]. Although off-label, encouraging results have emerged from the use of intravenous ketamine in treatment resistant OCD as reported by previous clinical studies and case reports [23–26], hence the growing interest in the use of intranasal esketamine in treatment resistant OCD.

The aims of this case report were to support the role of pharmacogenomic testing in psychiatry, especially in TRD patients, and to evaluate the effects of intranasal esketamine in the treatment of TRD with comorbid OCD.

## Case presentation

We hereby present the case of M.G., a 39-year-old male married engineer, who presented at our clinic for a major depressive episode in the context of a TRD. He had a positive psychiatric family history, since his mother suffered from MDD, while his father had an alcohol use disorder.

MDD onset in this patient had occurred at the age of 27, with a substantial recovery with the introduction of sertraline (50 mg/day) in combination with psychodynamic psychotherapy.

**Fig. 1:**
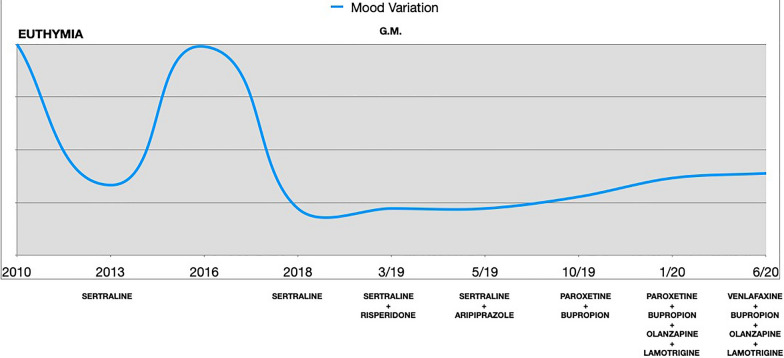
Mood variations from the first MDD episode to present

Despite a long disease-free period, in 2019, after his first son’s birth, M.G. experienced a relapse of MDD, characterized by a significant mood deflection, emotional lability, and severe fatigue. Hence, multiple pharmacological trials were made (Table 1), with only partial benefit (Fig. 1).

**Table 1:** Psychopharmacological history

Year	Medication	Dose	Duration	Notes
2013	Sertraline	50 mg	5 years	Recovery (primary treatment)
2018	Sertraline	200 mg	6 months	Partial response (primary treatment)
03/2019	Risperidone	2 mg	2 months	Stopped because of cognitive impairment (augmentation)
05/2019	Aripiprazole	5 mg	5 months	Stopped because of cognitive impairment (augmentation)
10/2019	Paroxetine	40 mg	6 months	No response (primary treatment, switched from Sertraline)
10/2019	Bupropion	300 mg	Ongoing	Partial response (combination)
01/2020	Olanzapine	5 mg	Ongoing	Partial response (combination)
01/2020	Lamotrigine	150 mg	Ongoing	Partial response (primary treatment)
06/2020	Venlafaxine	225 mg	Ongoing	Partial response (primary treatment)

In November 2020, due to the persistence of depressive symptoms, M.G. was referred to our Treatment-Resistant Disorders Clinic at San Gerardo Hospital, Monza, Italy by his private psychiatrist. During our first assessment, M.G. reported the persistence of anhedonia, low energy, asthenia, remarkable levels of anxiety and cognitive impairment (e.g.: persistent poor concentration and attentional deficits), which led to poor performance at work. The patient also reported to be emotionally detached from his family, friends, and environment.

Together with typical MDD symptoms, M.G. showed disabling symptoms related to OCD spectrum that led to the additional diagnosis of OCD according to the DSM-5 criteria [27, 28].

Indeed, over the last 2 years the patient had developed intrusive and egodystonic obsessions, consisting mainly in pathological doubts regarding his wife. Specifically, even if physically attracted to his partner, he felt forced to spend a considerable amount of time engaged in mental rituals, consisting of repetitively glancing at his partner to check her body features, such as her nose or chin and subsequently questioning himself about the meaning of these compulsions (e.g.: “Am I continuously checking her chin or nose because I don’t love her anymore?”, “I like her, so why do I have so many doubts about her?”). Such obsessive preoccupations, intrusive thoughts and rituals are what is commonly referred to as relationship obsessive–compulsive disorder [8, 29].

In addition, he also reported the presence of ritualistic intrusive and pervasive doubts regarding his mental health conditions. This implied the need to perform time-consuming mental rituals every morning (e.g.: independently from his psychopathological state, he used to repeat analytic checklists monitoring his conditions with precise order: “Am I feeling alright?”, “Am I depressed?”, “Am I happy?”, “Why did I cry?”, “Is it depression or something else?”, “If I feel I have little strength, does it mean that I am depressed?”).

When M.G. first came to our clinic, his therapy consisted of venlafaxine 225 mg/die, bupropion 300 mg/die, lamotrigine 150 mg/die, and olanzapine 5 mg/die.

In line with our Treatment-Resistant Disorders clinic protocol, clinical consultation, psychometric assessment (Table 2), Therapeutic Drug Monitoring (TDM) (Table 3) and Pharmacogenomic analysis were performed (Table 4).

**Table 2:** Psychometric assessment at first consultation

Rating scale	Score
BPRS	27
MADRS	15
YBOCS	15
CGI-S	3

*BPRS* Brief Psychiatric Rating Scale, *MADRS* Montgomery-Åsberg Depression Rating Scale, *YBOCS* Yale–Brown obsessive–compulsive scale, *CGI-S* clinical global impression-severity

**Table 3:** Therapeutic drug monitoring of venlafaxine at first consultation

Medication	Blood level	Therapeutic range*
Venlafaxine	484.5 ng/mL	100–400 ng/mL

Serum level of venlafaxine is measured as a level of venlafaxine active moiety (venlafaxine + 0-desmethylvenlafaxine). Method: LC–MS (Liquid chromatography–mass spectrometry). *According to Hiemke et al. [30]

**Table 4:** Pharmacodynamic (A) and pharmacokinetic (B) gene variations

Gene	Genotype
*(A)*	
SLC6A4 (rs63749047; rs25531)	S/S [Low activity]
MTHFR (rs1801133; rs1801131)	C677T: C/T A1298C: A/C [Low to intermediate activity]
ADRA2A (rs1800544)	C/G [Improved response]
HTR2A (rs7997012)	G/A [Normal response]
BDNF (rs6265)	Val/Val [Normal activity]
COMT (rs4680)	Val/Met [Normal activity]
HLA-A *31:01	Negative [Normal]
HLA-B *15:02	Negative [Normal]
DRD2 (rs1799732)	C/C [Normal activity]
MC4R (rs489693)	C/A [Normal activity]
5HT2C (rs3813929)	C/C [Standard weight gain risk]
ANK3 (rs10994336)	C/C [Normal activity]
CACNA1C (rs1006737)	G/G [Normal activity]
OPRM1 (rs1799971)	A/A [Normal activity]
GRIK1 (rs2832407)	A/A [Normal activity]
GABRA6 (rs3219151)	T/T [Increased risk]
GABRP (rs10036156)	T/T [Increased risk]
*(B)*	
CYP2B6	*4/*5 RM [High activity]
CYP2C19	*1/*17 RM [High activity]
CYP2D6	*2/*4 IM [Intermediate activity]
UGT2B15 (rs1902023)	*2/*2 IM [Decreased activity]
CYP1A2	*1A/H8 EM [Normal activity]
CYP2C9	*1/*1 EM [Normal activity]
CYP3A4	*1/*1 EM [Normal activity]
UGT1A4 (rs2011425)	*1a/*1a EM [Normal activity]
ABCB1 (rs2032583)	A/A [Normal activity]
ABCB1 (rs1045642)	G/G [Normal activity]

In the table pharmacodynamic as well as pharmacokinetic gene polymorphisms are listed along with a brief interpretation. *RM* rapid metabolizer, *EM* extensive (normal) metabolizer, *IM* intermediate metabolizer

Even though the patient resulted to have moderate depressive and obsessive symptomatology at psychometric evaluations, the patient’s quality of life was deeply affected, as he suffered from frequent crying fits, inability to concentrate at work and to engage in leisurable activities. Following the clinical interview, the patient was diagnosed with TRD with OCD symptoms and enrolled for intranasal esketamine treatment. A standard administration scheme was followed (Table 5), maintaining current patient treatment.

**Table 5:** Esketamine administration scheme

Phase	Period	Frequency of administration	Dose
Induction	First month	2 times/week	56 mg
Maintenance	Second month	1 time/week	56 mg
Maintenance	Third month and longer	1 time/2 weeks	56 mg

**Fig. 2:**
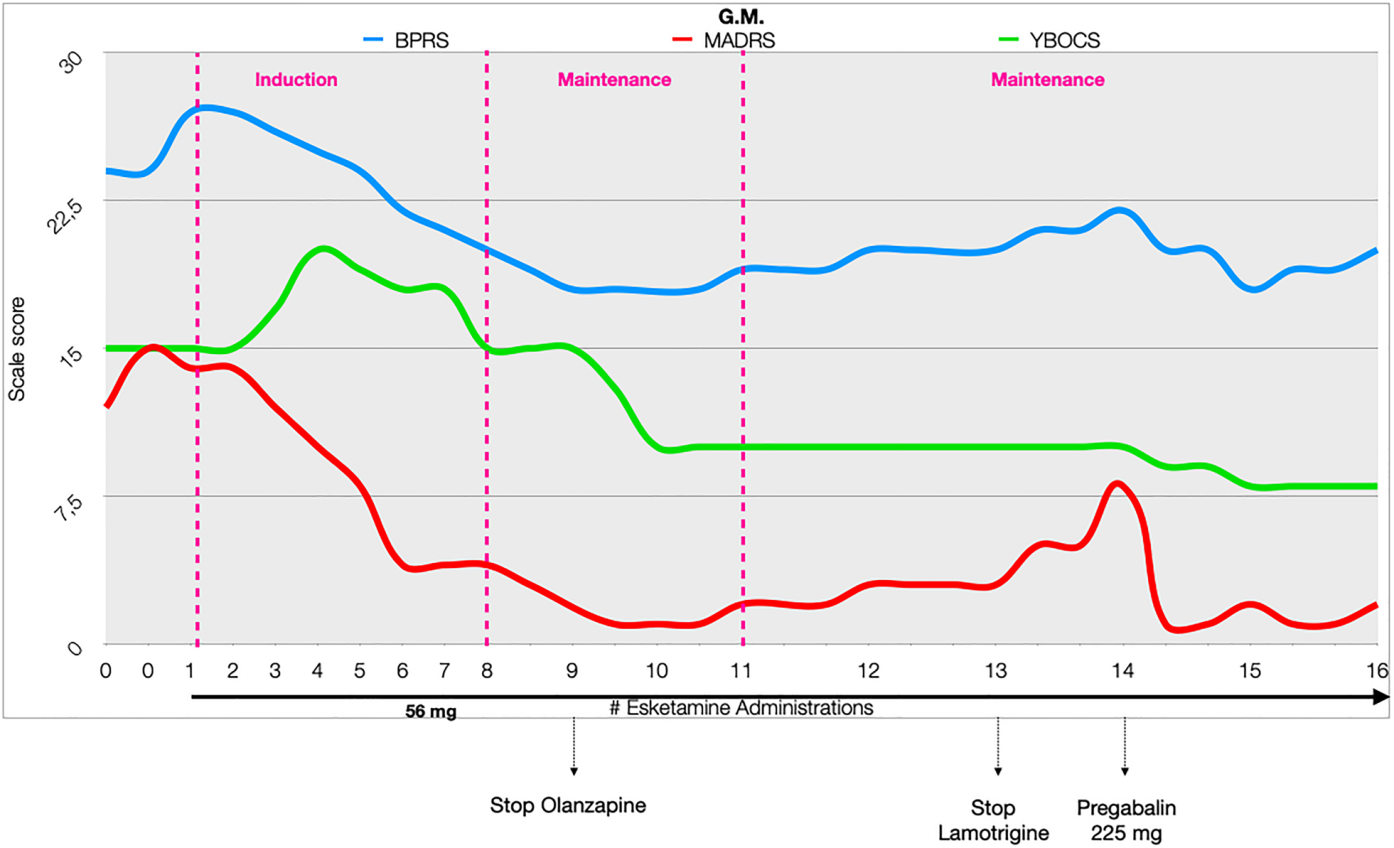
Variations of psychometric scales score. *BPRS* Brief Psychiatric Rating Scale,* MADRS* Montgomery-Åsberg Depression Rating Scale,* YBOCS* Yale–Brown obsessive–compulsive scale

As a result of esketamine introduction, the patient showed a rapid resolution of depressive symptoms during the induction phase and a significant reduction of OCD symptoms during the maintenance phase (Fig. 2).

By the time of the ninth esketamine administration, in consideration of the evident clinical improvement, olanzapine and lamotrigine were stopped after appropriate tapering. Some residual anxious symptoms were managed thanks to the introduction of pregabalin titrated up to 225 mg/die with further clinical benefit.

## Conclusions and discussion

The present case report emphasizes the need for thorough diagnostic investigation to optimize the management of treatment-resistant cases. Indeed, TDM and genetic profiling are of relevant importance to determine optimal treatment.

In this case, serum levels of venlafaxine were 484.4 ng/mL, which is above the therapeutic range according to the TDM guidelines in neuropsychopharmacology [30]. Thus, the abnormal biotransformation of the drug was not considered as an explanation for the treatment resistance.

Considering the results of pharmacogenomic analysis, the presence of 2 common single nucleotide polymorphisms in methylenetetrahydrofolate reductase (MTHFR) gene in this patient, specifically C677T and A1298C, might have contributed to his vulnerability to psychiatric disorders. MTHFR is an enzyme catalyzing the conversion of folic acid into its active form, methylfolate, which plays an essential role in monoamine biosynthesis [31]. T allele in C677T and C allele in A1298C might lead to decreased enzymatic activity, thus, as previously reported, an increased risk of affective disorders, such as MDD or other psychiatric disorders [32–34].

In addition, the genetic profiling also showed the S/S 5-HTTLPR genotype in SLC6A4 gene. SLC6A4 is a serotonin transporter responsible for serotonin reuptake. Such variations (*s* allele), previously described in the literature, are linked to decreased serotonin transporter expression in neurons, leading to higher susceptibility to depression, as well as poorer response to Selective serotonin reuptake inhibitors (SSRIs) [35–38]. This might also explain the patient's non-response to previous treatments with first-line antidepressants.

A prominent role in the brain control of stress plays GABA [39]. GABRA6 gene encodes the alpha6 subunit of GABA-A receptor and, according to previous studies, when exposed to recent negative stressful events, T allele carriers were at greater risk of depression- and anxiety-related symptoms which could also enhance suicidal risk [40]. Another study looked at the different types of recent life stressors and found that T/T genotype in patients, which was present in this case, interacts significantly with recent illness and personal problems stressors in influencing depression [41]. C allele carriers of another gene related to GABA, pi subunit of the GABA-A receptor (GABRP), were associated with good response to single antidepressant (either SSRI or venlafaxine) administered for at least 6 weeks [42]. In our patient the T/T genotype was found, representing a possible factor to his treatment refractoriness.

In addition, the patient was a CYP2B6 and CYP2C19 rapid metabolizer, potentially contributing to the inefficacy of bupropion and other SSRI. The predominant metabolic pathway of bupropion that leads to formation of its active metabolite hydroxybupropion is CYP2B6 enzyme-mediated. In case of rapid metabolizers, the therapeutic outcome of bupropion therapy is strongly affected [43].

Moreover, this case not only validates the rapidity of esketamine in reverting depressive symptoms, but it also shows encouraging findings about the possible use of esketamine in treating OCD symptoms.

Indeed, during the maintenance phase, the patient showed a significant reduction in his OCD symptomatology as showed by the Yale–Brown Obsessive–Compulsive Disorder (YBOCS) score reduction (Fig. 2, green line): after initial symptoms’ worsening due to a new-onset pathological doubt regarding treatment efficacy and side effects, the YBOCS score showed a 46.67% reduction (from 15 to 8). Since the pre-existing pharmacological treatment was not changed at our clinic, the reduction of the OCD symptoms might be directly referred to the use of esketamine.

It is worth noting that the time-ratio required to relieve OCD symptomatology maintained the 3:1 ratio usually seen with the use of serotonergic antidepressants [44, 45].

Literature regarding the possible use of ketamine in OCD remains sparse with some pre-clinical [46] and clinical studies [25, 26] showing a rapid reduction of OCD symptoms after the drug administration. Specifically, human studies indicated that ketamine could quickly and transiently decrease OCD behaviors. Nonetheless, these studies showed multiple limitations, mainly regarding small sample sizes and short-term observations. Thus, further investigation in the form of double-blind, randomized controlled trials is warranted.

## Abbreviations

MDD: Major depressive disorder
NMDA: *N*-Methyl-d-aspartate
OCD: Obsessive–compulsive disorder
PGx: Pharmacogenomic testing
SSRI: Selective serotonin reuptake inhibitors
TDM: Therapeutic drug monitoring
TRD: Treatment resistant depression
YBOCS: Yale–Brown obsessive–compulsive disorder

## Declarations

### Availability of data and materials

The data sets generated and/or analysed during the current study are not publicly available due to privacy reasons.
